# Potential to Use Fingerprints for Monitoring Therapeutic Levels of Isoniazid and Treatment Adherence

**DOI:** 10.1021/acsomega.2c01257

**Published:** 2022-04-21

**Authors:** Mahado Ismail, Catia Costa, Katherine Longman, Mark A. Chambers, Sarah Menzies, Melanie J. Bailey

**Affiliations:** † Department of Chemistry, University of Surrey, Guildford, Surrey GU2 7XH, U.K.; ‡ Ion Beam Centre, University of Surrey, Guildford, Surrey GU2 7XH, U.K.; § Faculty of Health and Medical Sciences, University of Surrey, Guildford, Surrey GU2 7XH, U.K.; ∥ Wexham Park Hospital, Frimley Health NHS Foundation Trust, Frimley, Surrey GU16 7UJ, U.K.

## Abstract

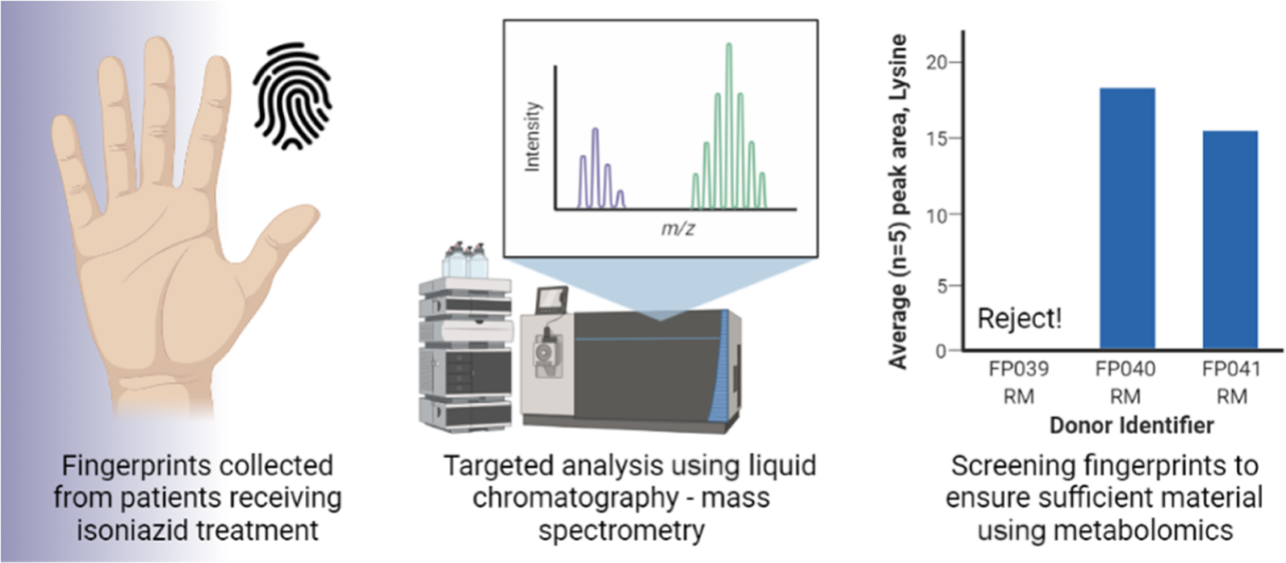

A fingerprint offers a convenient, noninvasive sampling matrix for monitoring therapeutic drug use. However, a barrier to widespread adoption of fingerprint sampling is the fact that the sample volume is uncontrolled. Fingerprint samples (*n* = 140) were collected from patients receiving the antibiotic isoniazid as part of their treatment, as well as from a drug-naive control group (*n* = 50). The fingerprint samples were analyzed for isoniazid (INH) and acetylisoniazid (AcINH), using liquid chromatography high-resolution mass spectrometry. The data set was analyzed retrospectively for metabolites known to be present in eccrine sweat. INH or AcINH was detected in 89% of the fingerprints collected from patients and in 0% of the fingerprints collected from the control group. Metabolites lysine, ornithine, pyroglutamic acid, and taurine were concurrently detected alongside INH/AcINH and were used to determine whether the fingerprint sample was sufficient for testing. Given a sufficient sample volume, the fingerprint test for INH use has sensitivity, specificity, and accuracy of 100%. Normalization to taurine was found to reduce intradonor variability. Fingerprints are a novel and noninvasive approach to monitor INH therapy. Metabolites can be used as internal markers to demonstrate a sufficient sample volume for testing and reduce intradonor variability.

## Introduction

For the first time in over a decade, tuberculosis (TB)-related deaths have increased.^1^ With increased disruption to medical services and reduced access to treatment, the ongoing pandemic of coronavirus disease (COVID-19) has reversed years of progress in the fight to end TB. As we enter the final year to achieve the historic targets set out in the first high-level United Nations (UN) meeting on TB in 2018, the need for effective treatment and disease management could not be more critical. It is estimated that in 2019, 10.0 million fell ill with TB, and of those, 465,000 cases were multidrug- or rifampicin-resistant TB (MDR-RR TB).^2^ Such cases arise when a patient is infected with *Mycobacterium tuberculosis* resistant to a particular antibiotic or where *M. tuberculosis* acquires resistance during treatment.^3,4^ Acquired resistance can develop through improper use of antibiotics (examples including poor adherence to a treatment regime or patients prematurely ceasing treatment) or due to suboptimal *in vivo* drug concentration in treatment regimens.^4,5^ The global success rate for treatment of drug-resistant TB is 57%, where treatment requires commitment to a regime of second-line drugs over a period of 9–20 months with continued counselling and support for adverse events.^2^

Treatment adherence in patients with TB is essential to limit transmission, avoid relapse, and restrict the development of drug resistance. Various strategies have been employed to increase adherence to treatment and improve success rates.^6,7^ Current methods to monitor adherence include regular engagement with medical and nursing teams, including provision of direct observation therapy (DOTS), tablet count, and assessment of clinical improvement.^8,9^ A urine dipstick test for the frontline TB drug, isoniazid (INH; based on a colorimetric test) can also be used on the spot if there are concerns or doubts regarding treatment efficacy.^10−12^ However, each of these approaches have their limitations: tablet count is ineffective because it is easily altered by the patient,^8,9^ engagement with the patient through DOTS is labor-intensive and expensive to administer,^9,13^ and a urine dipstick test requires clinic access.

Novel approaches for monitoring TB treatment adherence include the use of hair samples or saliva as a testing matrix.^14−17^ Hair can provide a snapshot of historic drug use (therapeutic or illicit), depending on the hair length and the stability of the drugs. However, it presents challenges with external contamination and/or effect of hair treatments (*e.g.*, hair dye), which can lead to false positive or negative results, respectively.^14^ Additionally, the quantitative potential of this matrix is poor, and publications have highlighted how failing to take historic medication uptake into consideration can result in false positive results or falsely high measurement of drug levels in hair samples.^14,16^ Saliva offers an alternative approach, but a recent publication highlighted issues with highly variable saliva–serum concentrations.^17^

In addition to adherence monitoring, it is of interest to clinicians to be able to carry out therapeutic drug monitoring to ensure that the drugs used to treat TB have been delivered at an appropriate therapeutic dose. The risk of multi-drug-resistant tuberculosis (MDR-TB) increases with suboptimal drug concentrations, which may arise due to nonadherence as well as other factors (malabsorption for example).^18−20^ This can be carried out by testing of venous blood samples using liquid chromatography mass spectrometry (LC–MS),^21−24^ or alternatively, the use of dried blood spots has been proposed.^25−27^ Collection of blood requires specialist equipment utilized by trained medical professionals, where additional complexities relating to necessary laboratory preprocessing steps for storage and transport can cause distress or discomfort in the donor. Such approaches are also prohibitively expensive for the majority of countries with the highest burden of infection.

A fingerprint, unlike blood, offers a unique opportunity for patients to provide samples noninvasively, both saving clinical resources and improving patient experience, as they are easily collected and transported. Furthermore, in a post-pandemic climate, the simplicity of fingerprint sampling could prove advantageous where access to clinics is limited, especially for high-risk individuals such as those with TB.

Prior research has shown the potential to use fingerprints for illicit drug testing^28−34^ and medical adherence monitoring.^35^ Despite a multitude of potential applications, uptake of fingerprint drug testing has been stunted by the ability to distinguish between drug use *versus* environmental exposure and the inability to determine, or control for, sample volume. The issue of possible contamination has been overcome through implementation of hand washing procedures,^28,36^ as well as application of appropriate cutoff levels.^31^ However, it is known that the collection of reproducible fingerprints remains highly challenging, where factors such as contact time, pressure, and time of collection can influence the amount and composition of material collected.^37^ Further, to control sample collection conditions, previous attempts have utilized endogenous compounds (*e.g.*, creatinine) to retrospectively adjust drug elimination profiles^29,30^ and mathematical modeling using metabolic biomarkers to account for variable sweat volume.^38^

Here, we describe for the first time the detection of INH and its metabolite, acetylisoniazid (AcINH), in fingerprint samples collected from patients undergoing treatment for TB. This provides proof of principle that a fingerprint sample can be used to monitor INH use. Additionally, we explore the variability in INH/AcINH and other endogenous compounds simultaneously detected in the fingerprint depositions from donors on INH treatment. We also demonstrate for the first time that endogenous markers can be used to verify that a sufficient fingerprint sample has been collected to eliminate false negative results.

## Materials and Methods

### Sample Collection

A favorable ethical opinion was obtained from the National Research Ethics Service (NRES—REC reference 16//LO/1663) for the collection and analysis of fingerprint samples taken from individuals receiving treatment for TB at Wexham Park Hospital (Frimley Health NHS Foundation Trust). Fingerprint samples (all five fingers of the right hand) were collected from (a) 28 patients (giving *n* = 140 samples) taking INH treatment for TB and (b) 10 individuals (giving *n* = 50 samples) who had never taken INH (negative control group). Samples were collected as per a previous work^31^ to include one set of samples “as presented” and another after handwashing. Fingerprint samples were collected on 2 × 2 cm squares of Whatman 1-Chr grade chromatography paper. Samples were collected using kitchen scales (Sainsbury’s Color) to measure the pressure applied during sample collection (800–1200 g for 10 s).

### Materials

INH, AcINH, and isoniazid-*d*_4_ (INH-*d*_4_; used as an internal standard) were purchased from Toronto Research Chemicals (North York, Canada). Optima-grade LC–MS solvents of methanol, acetonitrile (ACN), and water were used to prepare solutions and solvent mixtures (Fisher Scientific, Leicestershire, UK). Formic acid was added to the mobile phase solvents at 0.1% (v/v) (Fischer Scientific, Leicestershire, UK). Dichloromethane was used to prepare solvent extraction mixtures and was of analytical or reagent grade (Sigma-Aldrich, Dorset, UK). A solution of artificial eccrine perspiration was used to simulate a fingerprint matrix in the experimental design of fingerprint experiments (Pickering Laboratories, Inc., Obertaufkirchen, Germany).

### Sample Preparation and Analysis

Fingerprint samples were extracted as previously described.^31^ Extracted samples were reconstituted in 100 μL of 50:50 (v/v) ACN and water (with 0.1% formic acid) containing 50 ng/mL INH-*d*_4_ as the internal standard.

Chromatographic separation was performed on a Thermo Scientific Ultimate 3000 ultrahigh-performance LC (UHPLC) (Thermo Scientific, Bremen) using a Kinetex C18 column (100 × 2.1 mm, 5 μm) operated at 30 °C at a flow rate of 0.25 mL/min. Gradient analysis was performed with an initial mobile phase comprising 95% water (0.1% formic acid) and 5% ACN (0.1% formic acid), increased to 80% ACN (0.1% formic acid) and 20% water (0.1% formic acid) over 2 min, and kept constant for 0.5 min before returning to the initial mobile phase composition. The samples were introduced to a Q Exactive Plus Hybrid Quadrupole Orbitrap mass spectrometer (Thermo Scientific, Bremen) via the standard electrospray ionization (ESI) interface. Table S1 describes the ESI and MS parameters used for the analysis.

Analysis of 0.5–10 ng of INH and AcINH standards extracted from paper produced *R*^2^ values >0.999 and within a day precision of ≤3% (*n* = 5). The lower limit of detection was 10 pg for both analytes (see Figure S1). To evaluate matrix effects, 10 μL of the analyte standard at 100, 500, and 1000 ng/mL concentrations in methanol (equivalent to deposited masses of 1, 5, and 10 ng) was pipetted onto fingerprints (*n* = 5 donors) from nonpatients collected “as presented” and after handwashing, as well as a blank paper to monitor the response of AcINH and INH with and without fingerprints present. For all standards, the presence of a fingerprint suppressed the INH signal by ∼20%. This matrix effect varied by ≤9% for the five different donors. For AcINH, no overall signal suppression was observed, and the corresponding variability between donors was ≤14% (see Figure S2). A blank mobile phase injection was performed after each calibration standard in triplicate to evaluate the potential for carryover at each concentration level. No carryover was observed at any calibration level for INH, AcINH, or INH-*d*_4_. TraceFinder software (Thermo, UK) was used for retrospective interrogation of the data to search for compounds reported in previously published studies on MS applied to fingerprints.^28,37^ Peak assignment of sweat metabolites was carried out using the accurate mass of the protonated molecular ion (with a 3 ppm acceptance criterion). Samples extracted from a blank paper and analyzed using the same method were inspected to ensure that any compounds of interest were detected in the fingerprints at signal intensities above 3 times the levels measured in the blank samples. The metabolites investigated and their observed *m*/*z* values are given in Table S2.

## Results

Detection rates for INH analytes in participant fingerprints are summarized in Table 1. INH analytes were not detected in the fingerprints from the negative control group (Figure S3). AcINH was detected more frequently than INH, and 89% of fingerprints were positive for either INH or AcINH, reducing to 84% after handwashing. Based on these data, a fingerprint test for INH use performs better when both the parent drug and metabolite are used for detection, and handwashing should be avoided immediately prior to testing.

**Table 1:** Detection Rates of INH and AcINH from Individual Fingerprint Samples Collected from Patients (Taking INH Treatment) and the Negative Control Group (Not Taking INH Treatment)

		*n* (no. of fingerprint samples)	AcINH only (*n*, %)	INH only (*n*, %)	both analytes (*n*, %)	one analyte (*n*, %)	neither analyte (*n*, %)
unwashed hands	patients	140	29	1	95	124	15
			21%	1%	68%	89%	11%
	negative control	50	0	0	0	0	50
			0%	0%	0%	0%	100%
washed hands	patients	140	40	4	40	117	23
			29%	3%	29%	84%	16%
	negative control	50	0	0	0	0	50
			0%	0%	0%	0%	100%

The proportion of patient samples that were negative for both analytes was 11%, increasing to 16% after handwashing. There are several explanations for these negative results: (i) the patients had not taken their medication as directed, (ii) the sampling window exceeded the detection window of the drug, or (iii) an insufficient fingerprint sample had been deposited.

Despite efforts to control the fingerprint deposition parameters (deposition pressure, collection area, and contact time), a considerable intradonor range in the AcINH level was observed (Figure S4). This could arise from several factors: variation in volume of the fingerprint sample or variation from finger to finger in the concentration of the analyte.

**Figure 1:**
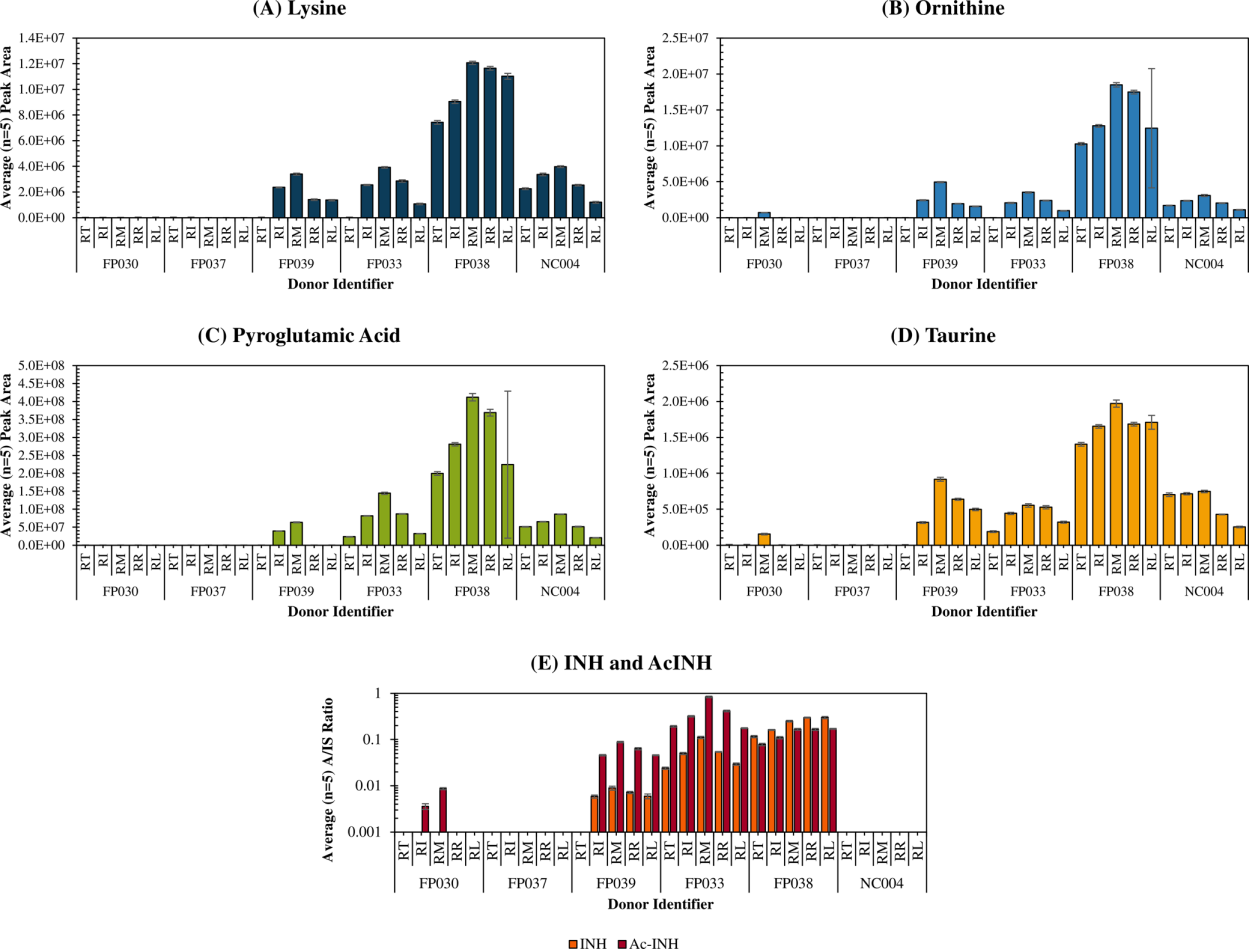
Average (*n* = 5, ±standard deviation) peak area of the extracted ion chromatogram for ions assigned to the protonated molecular mass of (A) lysine (*m*/*z* 147.1134), (B) ornithine (*m*/*z* 133.0977), (C) pyroglutamic acid (*m*/*z* 130.0504), and (D) taurine (*m*/*z* 126.0225) for a selection of fingerprint donors. FP037 and FP030 are examples of donors with poor INH detection, FP033 and FP038 are examples of good fingerprint donors, FP039 gives a mix of fingerprint depositions, and NC004 is a patient selected at random from the negative control group. Plot (E) shows the average (*n* = 5, ±standard deviation) analyte to internal standard (A/IS) ratio measured for INH and AcINH in the selected patient samples.

To decouple these possibilities, a retrospective inspection of the data was carried out to find biomarkers of fingerprint sweat. A range of analytes previously reported in fingerprints and eccrine sweat were considered (Table S2). Of these, protonated molecular ions corresponding to the amino acid or amino acid derivatives lysine, ornithine, pyroglutamic acid and taurine were most frequently detected, and Figure 1 shows the intradonor variation in these compounds for a selection of donors. Figure 1 clearly shows a relationship between the detection of endogenous metabolites and the detection of AcINH. For example, AcINH was detected in all fingerprints of donors FP033 and FP038, as well as pyroglutamic acid and taurine. Nondetection of AcINH from any fingerprint of donor FP037 coincided with the nondetection of the candidate eccrine sweat markers, which suggests that an insufficient fingerprint material was collected. Therefore, it cannot be determined if the participant had taken INH as the sample volume is insufficient. For donors FP030 and FP039, detection of AcINH in only some donated fingerprints corresponded well with the detection of eccrine sweat markers. NC004 was chosen at random from the negative control group to demonstrate that eccrine sweat markers are not only associated with INH use.

**Figure 2:**
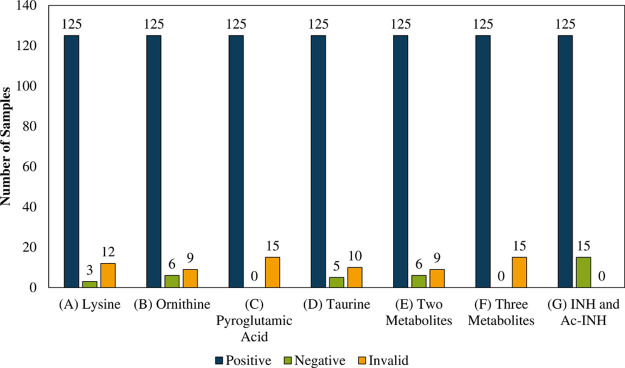
Number of patient samples testing positive for INH use (blue, based on INH or AcINH); negative for INH use (green, based on INH or AcINH); and invalid test (yellow) based on the criteria of detection of one or a combination of the selected four sweat metabolites.

The results in Figure 1 provide evidence that detection of INH/AcINH typically coincides with the detection of eccrine sweat metabolite markers. We propose that these compounds can therefore be used as internal markers to determine whether the fingerprint sample volume was sufficient for INH testing to take place. The detection rate for INH use was recalculated based on a requirement for the simultaneous detection of various sweat metabolites and combinations thereof. Figure 2 shows the performance of six candidate testing protocols, under which a fingerprint sample should contain lysine (A); ornithine (B); pyroglutamic acid (C); taurine (D); ≥2 metabolites (E); and ≥3 metabolites (F) to be considered a valid sample, benchmarked against a test based on the detection of INH/AcINH only (G). Test protocols (C) and (F) resulted in the elimination of all false negatives, with <11% of samples being classed as invalid.

The performance (sensitivity, precision, specificity, and accuracy) of fingerprint testing protocols C, F, and G is shown in Table 2. Detection based on pyroglutamic acid (protocol C) or three amino acids (protocol F) showed improved performance when compared to detection of INH/AcINH alone (protocol G).

**Table 2:** Calculated Accuracy, Precision, Sensitivity, and Specificity for INH Use Using the Criteria of Detection of Pyroglutamic Acid (Test Protocol C), Three Amino Acids (from Lysine, Ornithine, Pyroglutamic Acid, and Taurine; Test Protocol F), or None (Test Protocol G)

	detection based on pyroglutamic acid (C) (%)	detection based on 3 metabolites (F) (%)	detection based on INH and/or AcINH (G) (%)
accuracy	100	100	92
precision	0	0	8
sensitivity	100	100	100
specificity	100	100	77

**Figure 3:**
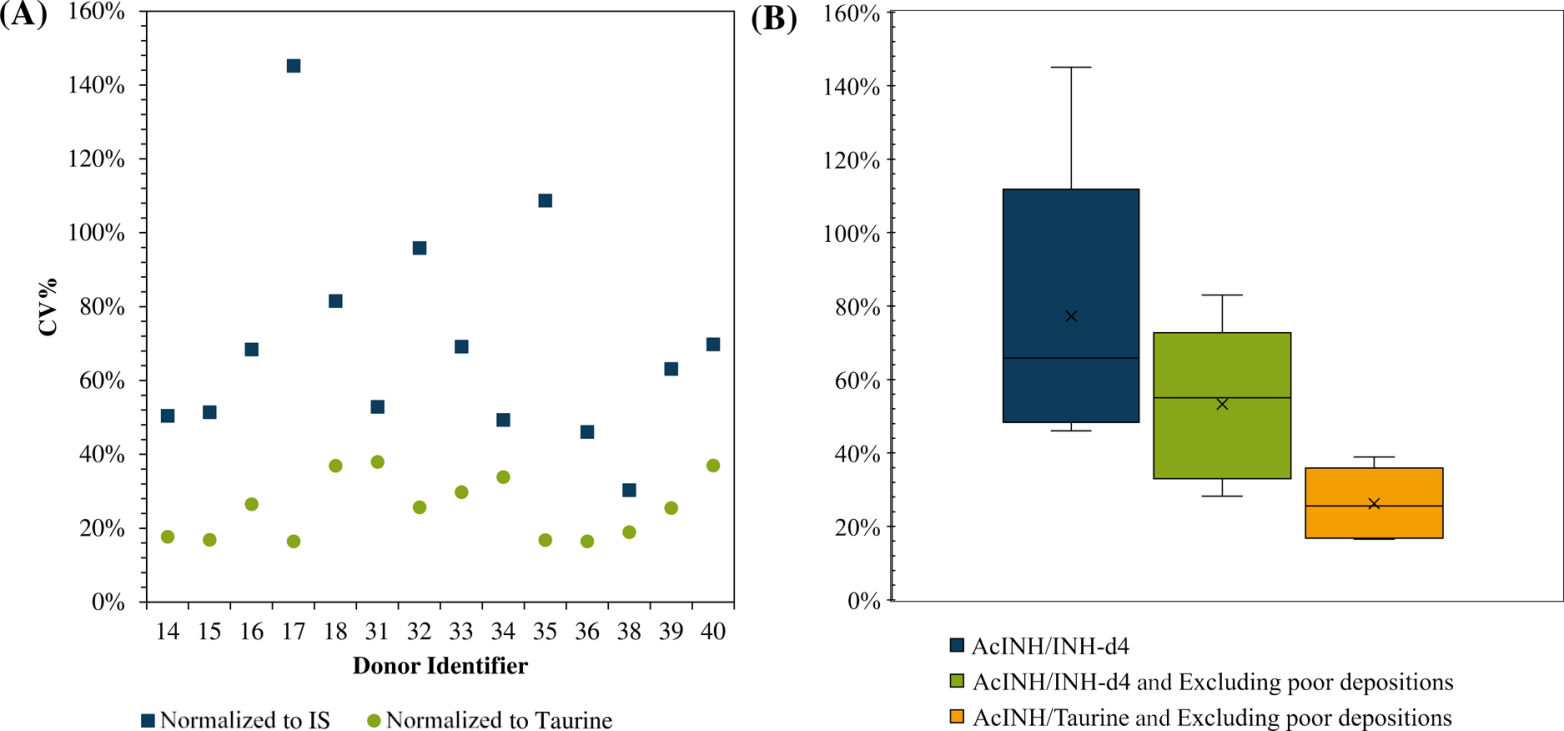
(A) Comparison per donor of the CV of AcINH measurements from fingerprints using only the internal standard for normalization (blue) and using normalization to taurine (orange). (B) Box plots showing the distribution (range, upper and lower quartiles, and median) of CVs obtained for the different fingerprint sets using different normalization strategies.

In addition to identifying samples for which insufficient fingerprint material was collected, this work shows that the metabolite signals can also be used for data normalization to control for the volume of the material deposited. Figure S4 shows the considerable intradonor variability in AcINH per fingerprint. In Figure 3A, samples in which the metabolite taurine was not detected are rejected to exclude poor depositions. The AcINH signal was then normalized to taurine. In Figure 3A, the coefficient of variation (CV) of fingerprint samples deposited by the same donor are plotted after internal standard normalization (blue) and taurine normalization (green). Normalizing to taurine reduces the CV for every donor.

In Figure 3B, box plots depicting the CVs of fingerprint sample sets are presented with different normalization strategies. The box plot on the left depicts the CVs of the fingerprint sample sets produced by normalizing only to the internal standard and without any consideration for the quality of the fingerprint. The middle box plot shows the same data, but now rejecting samples for which taurine was not detected. The far-right box plot shows the effect of additionally normalizing to taurine. The median CV reduces from 65 to 55 and then to 26% using these normalization strategies. Normalizing to the other endogenous compounds was found to improve the CV with inconsistent results (Figure S5).

## Discussion

This work has demonstrated how markers of eccrine sweat can significantly improve fingerprint drug testing by highlighting and rejecting samples for which insufficient material has been deposited. This approach resulted in sensitivity, specificity, and accuracy of 100% but resulted in 11% of fingerprint samples (*n* = 15) being inadequate for testing purposes. Of the 28 patients (*n* = 140 fingerprint samples), only two patients returned invalid tests for all five collected fingerprints (FP037 and FP041). The right index and right middle fingers were the only ones that did not return any invalid results for any patient. Future work should explore increasing the number of viable samples by exploring methods to enhance the deposition of fingerprint residues, for example, by increasing the deposition time and/or contact pressure or application of more sensitive analytical methods.

Finally, the ability to relate the level of the drug deposited in a fingerprint to the blood plasma level would provide much broader opportunities for fingerprint-based diagnostics. Variation in the mass of the drug or metabolite per fingerprint across a set of fingerprints currently poses a barrier to any meaningful comparison of fingerprint samples with any other biofluid. This work also showed that normalizing AcINH to amino acid taurine can be valuable in reducing the intradonor variability across a set of deposited fingerprints. This is a first step toward providing a quantitative test from a fingerprint, and future studies should explore whether the intradonor variability can be reduced further through targeted analysis of taurine and the other fingerprint biomarkers identified here. A method to standardize or account for the mass of fingerprint deposition could enable quantitative analysis with future applications, including the potential for reliable monitoring of antibiotics in the fingerprints of patients undergoing treatment for TB and other infectious diseases.

## Conclusions

INH and AcINH can be detected in natural fingerprints from patients receiving TB treatment. Our findings show that a fingerprint test for INH use should test for both INH and its metabolite AcINH, and handwashing should be avoided immediately prior to testing. Additionally, markers of eccrine sweat can be used to monitor whether sufficient fingerprint has been collected and reduce intradonor variability. The test protocol based on eccrine sweat markers developed to assure the sample quality gave an accuracy of 100%.

## Supporting Information Available

Additional instrumental conditions, calibration curves, theoretical and observed molecular masses, matrix effect experimental results, and study participant results (PDF)
