# Supplementary material for: Potential to Use Fingerprints for Monitoring Therapeutic Levels of Isoniazid and Treatment Adherence
Source: ACS Omega. 2022 Apr 21;7(17):15167–73. doi: 10.1021/acsomega.2c01257 (PMC9089686; doi:10.1021/acsomega.2c01257)
Supplement: Supplementary file 1 — ao2c01257_si_001.pdf [file ao2c01257_si_001.pdf]

The potential to use fingerprints for monitoring therapeutic levels of isoniazid and treatment adherence

Mahado Ismail[1], Catia Costa[2], Katherine Longman[1], Mark A. Chambers[3], Sarah Menzies[4], Melanie J. Bailey[1*]

[1] University of Surrey, Department of Chemistry, Surrey, GU2 7XH, UK; [2] University of Surrey Ion Beam Centre, Surrey, GU2 7XH, UK; [3] University of Surrey Faculty of Health and Medical Sciences, Surrey, GU2 7AL, UK; [4] Wexham Park Hospital, Frimley Health NHS Foundation Trust, Slough, SL2 4HL

* Address corresponding to this author at: University of Surrey, Department of Chemistry, Surrey, GU2 7XH, UK. E-mail: m.bailey@surrey.ac.uk

Table S1: Operating conditions of the LC-MS for the analysis of isoniazid and acetylisoniazid in fingerprint samples.

**Parameter**	**Operating condition**
Source parameters	
Spray voltage	4 kV
Capillary temperature	320 ºC
S-lens RF level	50
Sheath gas flow rate	35
Aux gas flow rate	8
Scan parameters	
Scan type	Full MS
Scan range	*m/z* 120 - 1000
Resolution	70 000 at *m/z* 200
Polarity	Positive
AGC target	10^6
Maximum inject time	200

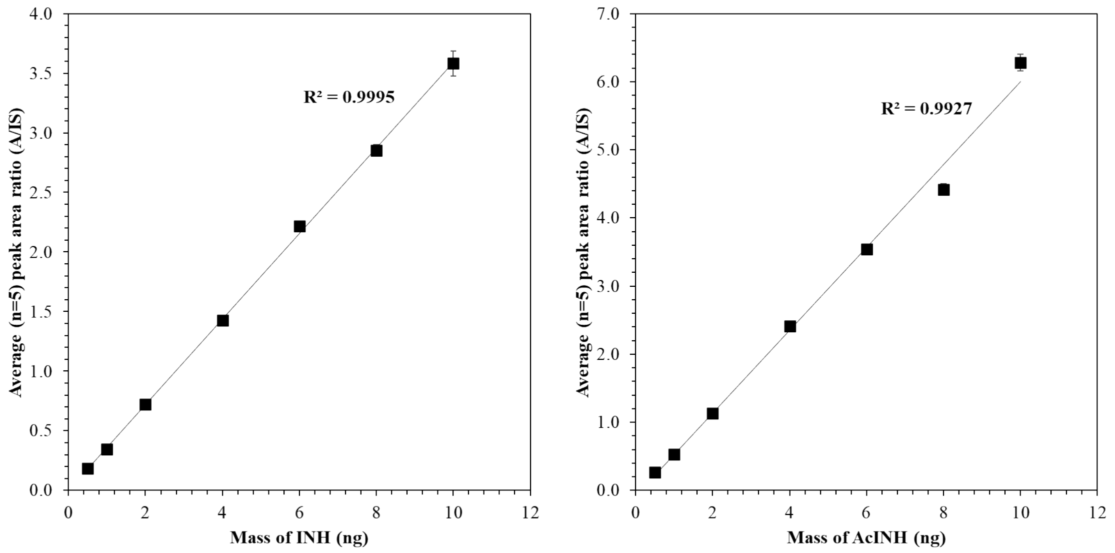

Figure S1: Calibration curve of isoniazid and acetylisoniazid ranging from 0.5 – 10 ng of extracted analyte on surface analysed using LC-MS.

Table S2: Metabolite peak assignment, m/z values observed in fingerprint samples, theoretical m/z and mass deviation based on the protonated molecular ion.

Assignment	Observed *m/z*	Theoretical *m/z*	Error (ppm)
Pyroglutamic Acid	130.05	130.0499	0.77
Ornithine	133.0972	133.0972	0.00
Taurine	126.022	126.0219	0.79
Histidine	156.0773	156.0768	3.20
Leucine	132.1019	132.1019	0.00
Threonine	120.0661	120.0655	5.00
Arginine	175.1195	175.119	2.86
Asparagine	133.0608	133.0608	0.00
Aspartic Acid	134.0453	134.0448	3.73

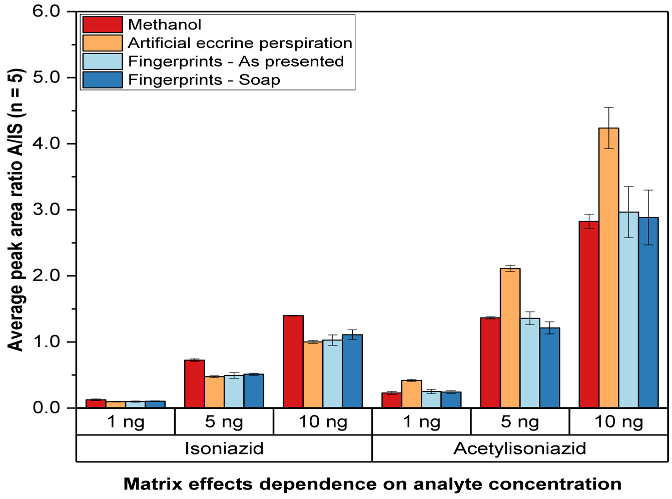

Figure S2: Average peak area ratio analyte (A) to internal standard (IS) (± standard deviation, n = 5 measurements) for isoniazid and acetylisoniazid for spiked methanol, artificial eccrine perspiration and fingerprints (as presented and after washing hands with soap).

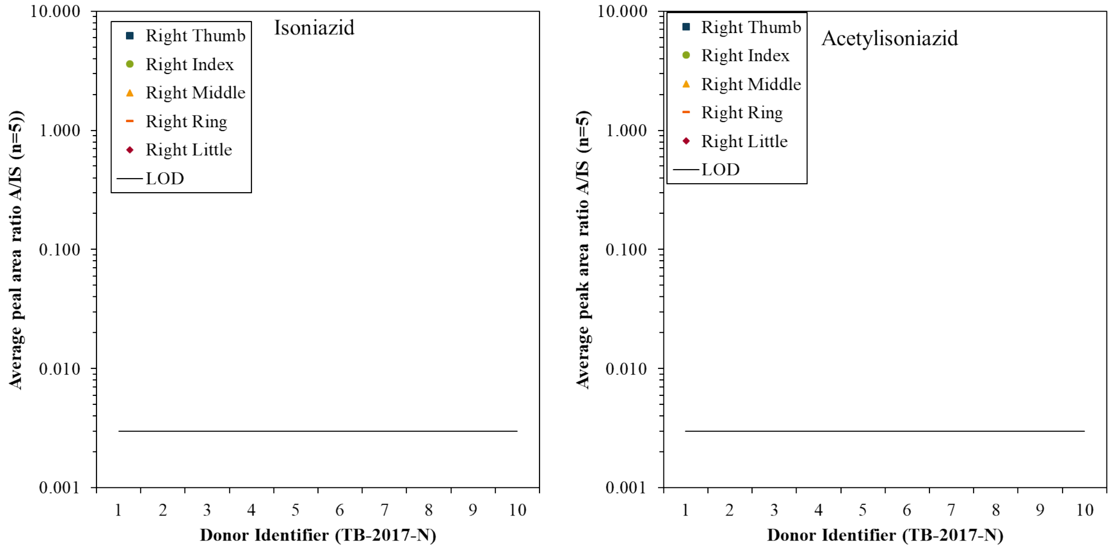

Figure S3: Fingerprint results (average peak area ratio analyte (A) to internal standard (IS), ± standard deviation n = 5 measurements) for isoniazid and acetylisoniazid in fingerprint samples collected as presented for (n = 10) participants as negative control subjects. Solid line represents limit of detection (LOD).

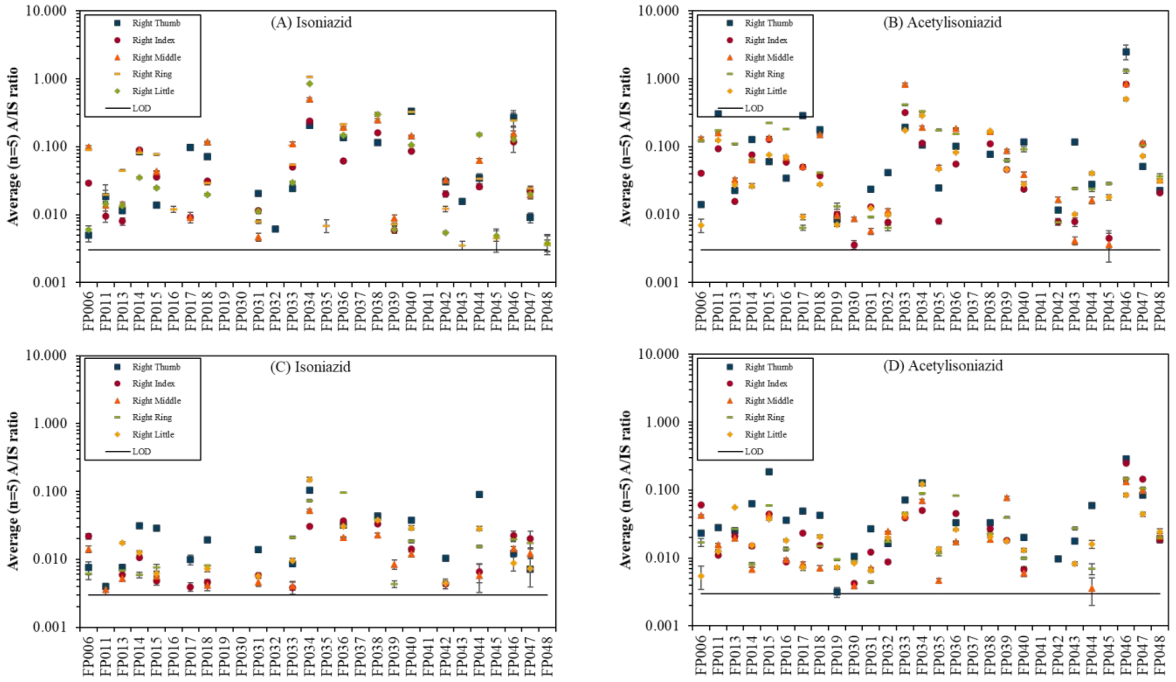

Figure S4: Average peak area ratio of analyte to internal standard (A/IS), ± standard deviation (n = 5 injections) for isoniazid (A and C) and acetylisoniazid (B and D) in fingerprint samples collected as presented (A and B) and after washing hands (C and D) and analysed using LC-HRMS for 28 participants on treatment for tuberculosis. Line represents limit of detection (LOD). Y-axis on log10 scale.

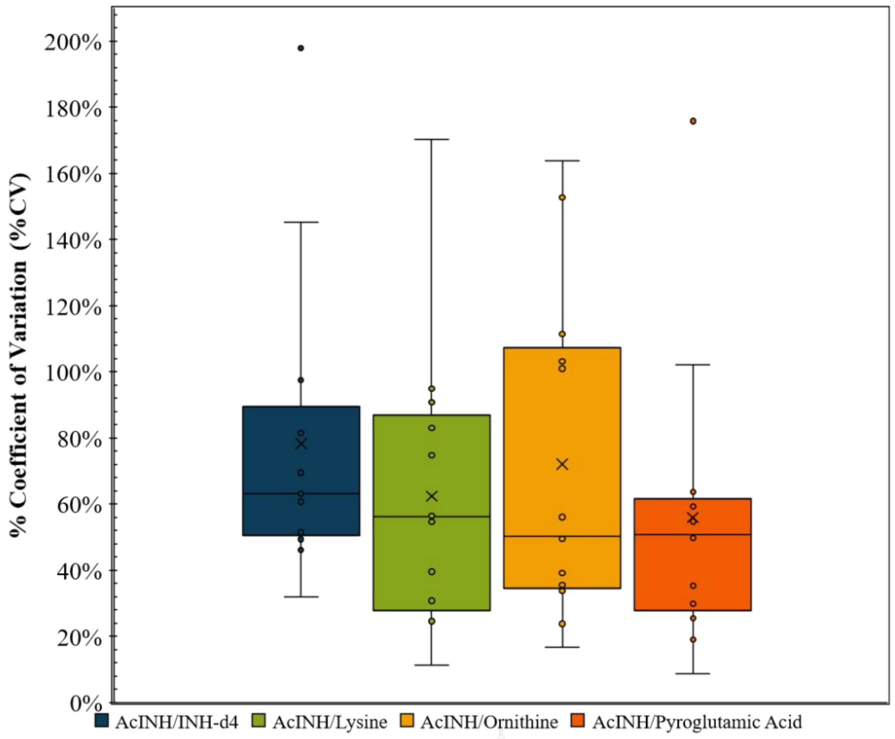

Figure S5: Box plots showing the distribution (range, upper and lower quartile and median) of % coefficient of variation (CV) obtained for the different fingerprint sets using different normalisation strategies: acetylisonaizid normalised to ions assigned to the internal standard (IS; INH-D4), lysine, ornithine and pyroglutamic acid for different patient sample sets.
